# Seronegative Celiac Disease - A Challenging Case

**DOI:** 10.7759/cureus.27730

**Published:** 2022-08-06

**Authors:** Inês M Araújo, Margarida Robalo, Rui Domingues, Marta Viana Pereira, Sofia Esperança

**Affiliations:** 1 Internal Medicine, Braga Public Hospital, Braga, PRT; 2 Medical Oncology, Braga Public Hospital, Braga, PRT

**Keywords:** gluten-free diet, diagnosis, villous atrophy, celiac disease, nausea and vomiting

## Abstract

Celiac disease is an inflammatory disorder of the small intestine caused by sensitivity to gluten. This enteropathy results from the interaction between genetics, autoimmunity, and an environmental trigger (gluten). It can manifest at all ages. We present a case of a 76-year-old woman with nausea and vomiting for six months. She reported asthenia, weight loss, and a brief period of diarrhea without blood or mucus. The search for evidence of infection, tumours, and endocrinopathies was negative, as well as the immunological study, including antibodies for celiac disease. Upper endoscopy with biopsies revealed villous atrophy. Capsule endoscopy showed macroscopic features suggestive of celiac/Whipple’s disease. Duodenal biopsies were reviewed, and Whipple’s disease was considered unlikely. The genetic analysis was positive for HLA DR7-DQ2. After one year on a gluten-free diet, there was a clinical and histological improvement. The diagnosis of seronegative celiac disease is complex and requires the exclusion of other causes of villous atrophy, as well as a histological improvement after one year of treatment. The genetic test has a high negative predictive value.

## Introduction

Celiac disease is based on the immunological response to an environmental trigger (gluten) in genetically predisposed individuals. This inflammatory enteropathy caused by sensitivity to dietary gluten is one of the most common autoimmune disorders. It is more common in females and can present at any age [1,2].^ ^It is worth noting the increase seen in recent years in its incidence in individuals over 65 years of age [3].

Regarding the clinical presentation, it is divided into intestinal and extraintestinal symptoms. It is classically characterized by diarrhea and malabsorption, which can lead to weight loss and asthenia. Hypoalbuminemia can be present in severe cases. The absorption of vitamin B12, folic acid, iron, calcium, and vitamin D may be compromised, leading to anemia and osteoporosis [1,2].^ ^Alternation with constipation and the presence of nausea and vomiting are less common and most seen when this disease presents for the first time in adulthood. Extraintestinal manifestations include dermatitis herpetiformis, aphthous stomatitis, dental enamel hypoplasia, peripheral neuropathy, ataxia, depression and anxiety, elevation of transaminases, and pancreatitis [3].^ ^Its association with other autoimmune pathologies is common [1,2].

Due to its many clinical manifestations, including non-specific symptoms, the diagnosis of celiac disease can be challenging. The authors presented a case that was difficult to manage, describing the structured diagnostic approach conducted and the success of the treatment after achieving a diagnosis. 

## Case presentation

This clinical case refers to a 76-years-old woman who presented for evaluation in the emergency department with symptoms of nausea and vomiting for the past six months. She was feeling asthenic and had lost twelve kilograms in that period of time. In addition, she mentioned two months before the present evaluation a brief period of diarrhea without blood or mucus. The patient denied fever, adenopathies, cutaneous manifestations, arthralgias night sweats, and respiratory or genitourinary symptoms. She had no previous contact with other ill people or with animals and had not traveled abroad recently. Regarding to food, she had no dietary restrictions and denied consumption of unpasteurized dairy products, and drank tap water. She did not smoke and denied consumption of alcohol, drugs, supplements, or herbal products.

Regarding to patient’s medical history, the authors highlight the diagnosis of hypertension medicated with olmesartan 20 mg and amlodipine 5 mg daily. On evaluation at the emergency department, the patient was hemodynamically stable, conscious, oriented, pale, and emaciated. On physical examination, there was evidence of mild discomfort on abdominal evaluation, but without signs of peritoneal irritation, palpable masses, or organomegaly. The laboratory workup detected the presence of anemia, hypoalbuminemia, mild cytolysis, and cholestasis (Table 1). The patient was admitted to the internal medicine service for study and symptom management.

**Table 1:** Laboratory workup at the emergency department

Analysis	Result (reference value)	Analysis	Result (reference value)
Hemoglobin	8.4 g/dL (11.9-15.6 g/dL)	Alkaline phosphatase	140 U/L (46-120 U/L)
Leucocytes	4100 /uL (4000-11000)	Aspartate aminotransferase	45 U/L (12-40 U/L)
Platelets	246000 /uL (150000-4000/uL)	Alanine Aminotransferase	51 U/L (7-40 U/L)
Urea	27mg/dL (19.49 mg/dL)	Amylase	72 U/L (<125 U7L)
Creatinine	0.6 mg/dL /0.6-1.2 mg/dL)	Lipase	23 U/L (<160 U/L)
Potassium	4.1 mmol/L (3.5-5.1 mmol/L)	C-reactive protein	9.9 mg/dL (<5 mg/dL)
Sodium	133 mmol/L (136-145 mmol/L)	Sedimentation rate	13 g/dL (<15 g/dL)
Total bilirubin	1.54 mg/dL (0.3-1.2 mg/dL)	Albumin	2.2 g/dL (3.4-5) g/dL
Direct bilirubin	0.99 mg/dL (<0.3 mg/dL)	Serum protein electrophoresis	Normal

The study carried out so far was further explored during hospitalization. Five months before the current episode, a computed tomography (CT) of the abdomen and pelvis was performed, which revealed some distension of the colon; however, there was no evidence of obstruction. Three months before the current evaluation, an upper digestive endoscopy with gastric biopsies revealed chronic gastritis with lymphoplasmacytic infiltrate. There was no reference to duodenal biopsies. Colonoscopy was also performed, with no abnormalities detected.

Gastrointestinal infections are one of the most common causes of nausea and vomiting. However, this patient did not present with an increase in inflammatory markers favoring this hypothesis. Hepatitis and human immunodeficiency virus serologies were also negative (Table 2). Parasitological analysis of stools and serology for *Echinococcus granulosus* and *Fasciola hepatica* were negative. The most frequent zoonoses were also excluded.

**Table 2:** Laboratory workup during hospitalization ACTH - adrenocorticotropic hormone; PTH - parathyroid hormone; T4 - thyroxine; TSH - thyroid stimulating hormone; anti-HAV - hepatitis A virus antibodies; Ig - immunoglobulin; anti-HBs - hepatitis B surface antibody; HBs antigen - hepatitis B surface antigen; anti-HCV - hepatitis C virus antibodies; anti-HIV - human immunodeficiency virus antibodies

Analysis	Result (reference value)	Analysis	Result (reference value)
TSH	2,311 uUI/mL (0.6-4.8 uUI/mL)	HBs Antigen	Negative
Free T4	1,23 ng/dL (0.9-1.8 ng/dL)	Anti-HBs	Negative
PTH	72,45 pg/mL (10-55 pg/mL)	Anti-HCV	Negative
Cortisol	20,46 ug/dL (6-23 pg/mL)	Anti-HIV I/II	Negative
ACTH	16,90 pg/mL (10-60 pg/mL)	IgA	276 mg/dL (40-350 mg/dL)
Aldosterone	9,61 ng/dL (6.8-17-3 ng/dL)	Anti-endomysium antibodies	Negative (1/5)
Anti-HAV (IgG)	Positive	Anti-transglutaminase antibodies	2.5 AU/mL (<10 AU/mL)
Anti-HAV (IgM)	Negative	Anti-gliadin antibodies	IgA 1 UI/mL (<10 UI/mL); IgG 0 UI/mL (<10 UI/mL)

Endocrinological pathologies such as diabetes, hyper/hypoparathyroidism, hyperthyroidism, and adrenal insufficiency that can also lead to nausea and vomiting were excluded (Table 2).

Concerning neurological causes, a cranioencephalic CT was performed, which proved to be normal. The patient did not show any mood changes, calluses on the back of the hands, or parotid hypertrophy that would raise the suspicion of a psychiatric cause.

Given the patient’s age, tumours appear as a mandatory differential diagnosis. Previous imaging tests were not suggestive of malignancy. Even so, as there was a progressive worsening of cytocholestasis during hospitalization, magnetic resonance cholangiopancreatography was performed, which only revealed the presence of biliary sludge without pancreatic or bile duct changes. During hospitalization, right ilio-femoro-popliteal deep vein thrombosis was observed, which increased the suspicion of a neoplastic etiology contributing to the hypercoagulable state. However, positron emission tomography was performed, with no evidence of anomalous uptake.

Regarding toxic causes, the measurement of heavy metals was negative. The patient was under therapeutic with olmesartan, which leads to the hypothesis of olmesartan enteropathy. This is an exclusion diagnosis; however, given the presence of compatible symptoms, the drug was discontinued at the time of admission. Despite the drug suspension, the patient maintained food intolerance, requiring the introduction of parenteral nutrition.

Considering the remote hypothesis of a neuroendocrine tumor, the urinary excretion of 5-hydroxyindoleacetic acid (5-HIAA) was measured, and somatostatin receptor scintigraphy was performed. Both tests revealed normal results.

At this time, it was decided to repeat the upper digestive endoscopy. Duodenal biopsies revealed villous atrophy. Serologies for celiac disease (anti-transglutaminase, anti-endomysium, and anti-gliadin antibodies) were negative (Table 2), so a genetic study was requested.

Given the evidence of lymphoplasmacytic infiltrate in the gastric biopsy performed before the current admission, in association with the cytocholestatic alterations found in the analyses, IgG4 disease was considered. Measurement of serum IgG4 was reduced, and the histopathological analysis of the new biopsies excluded this diagnosis. The remaining immunological study was negative.

**Figure 1:**
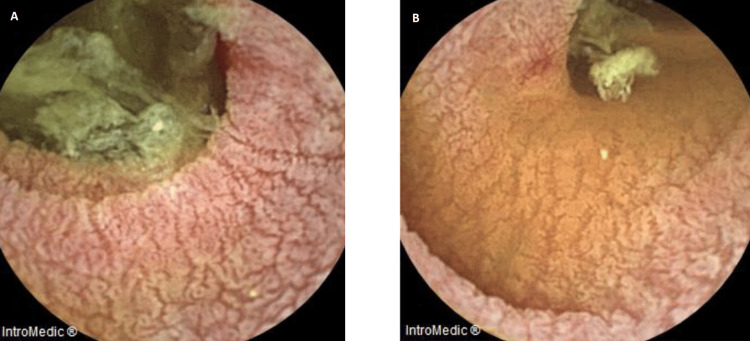
Images obtained by capsule endoscopy Along the small intestine, there is a cleft, whitish and swollen mucosa (A). Some erosions and angiectasia are present at the level of the middle and distal jejunum and the proximal ileum (B). The findings described are consistent with celiac disease/Whipple’s disease.

The use of capsule endoscopy allowed for a better clarification, revealing a whitish edematous mucosa (Figure 1), erosions, and angiectasia, which are findings compatible with celiac disease/Whipple’s disease. 

Given the severity of the patient’s clinical condition, it was decided to start antibiotic therapy with ceftriaxone and simultaneously start a gluten-free diet as tolerated. A marked clinical improvement was evident. After two weeks, neurological manifestations of Whipple’s disease had been excluded, and antibiotic therapy was changed to co-trimoxazole. Posteriorly, it was possible to review the biopsied fragments, and both the search for macrophages positive for periodic acid-Schiff and the immunohistochemistry for *Tropheryma whipplei* were negative. The polymerase chain reaction (PCR) to detect bacterial DNA in serum was also negative. This way, this diagnostic hypothesis became unlikely.

The genetic study revealed positivity for HLA DR7-DQ2, with HLA DR7-DQ8 being negative. Thus, seronegative celiac disease stands out as the most likely diagnosis in this case.

The patient’s first clinical reassessment took place after two months on a gluten-free diet. The patient was asymptomatic, and both anemia and cholestasis were improving. After one year, upper endoscopy with biopsies was repeated, and histological recovery was evident, confirming the final diagnosis.

## Discussion

Olmesartan enteropathy courses with abdominal pain and distension, vomiting, and diarrhea and may cause hypoalbuminemia and anemia. The median time from drug exposure to symptom development is 3.1 years. It affects both men and women. Endoscopic studies typically reveal nodular intestinal mucosa, villous atrophy, and wall ulceration. As observed in this case, failure to improve within two weeks after drug discontinuation decreases the likelihood of olmesartan being the cause [4,5].

Whipple’s disease is caused by infection with *Tropheryma whipplei* and affects mostly men [6]. The first symptom to appear is usually arthralgia, followed by gastrointestinal symptoms. In addition, low-grade fever, asthenia, and weight loss may be associated. Pulmonary, cardiac, and neurological affection may also occur. Upper digestive endoscopy with small intestine biopsies allows the identification of macrophages positive for periodic acid-Schiff staining and lipid invasion of the lamina propria. It may be possible to identify the bacteria by electron microscopy [7]. None of these features were present in this clinical case.

Celiac disease could cause a spectrum of clinical features that extend from mild nutritional deficiencies to life-threatening severe malabsorption. As described in the literature, some patients may even need supportive parental nutrition, as seen in this case [8]. Nevertheless, presentation with nausea and vomiting is less common, contributing to the complexity of this case. 

There is a known increased incidence of celiac disease among elderly people. This case is in agreement with the delay in diagnosis described in the literature when there is a late onset of symptoms [9].

Diagnosis of celiac disease implies the presence of at least four of the following criteria: 1) diarrhea and malabsorption, 2) positive antibodies, 3) positivity for HLA-DQ2 and/or HLA-DQ8, 4) intestinal mucosal damage, 5) clinical response to a gluten-free diet [1].

This patient had symptoms consistent with the diagnosis and positivity for HLA DR7-DQ2. In addition, duodenal biopsies revealed villous atrophy, a known feature of celiac disease. The fourth criterion was achieved with symptom improvement after the implementation of a gluten-free diet.

In the face of clinical suspicion, the IgA anti-transglutaminase antibody, which is the most sensitive antibody, should be measured. If its presence is positive, the anti-endomysium antibody should be obtained for confirmation, as it is more specific. It is essential to simultaneously exclude the IgA deficit that may be associated with celiac disease. When IgA deficit is present, IgG anti-gliadin antibody should be the one to measure instead. When serologies are positive, there is an indication for duodenal biopsy [2].

Upper digestive endoscopy is recommended to obtain four biopsies in the second portion of the duodenum and two in the bulb. Villous atrophy, crypt hyperplasia, and the presence of intraepithelial lymphocytes are the typical histological features of celiac disease [2].

Serologies are negative in a small percentage of patients (2-3%), mostly female, a case that is associated with a higher risk of refractoriness to treatment and greater morbidity and mortality [1]. In these cases, if clinical suspicion is high, testing for HLA-DQ2/HLA-DQ8 is essential because a negative result will exclude the diagnosis. If the presence of one of them is confirmed, a gluten-free diet should be introduced [10]. The case presented refers to a woman that belongs to that 2-3% of people with negative antibodies, which can lead to a delay in the diagnosis, affecting the disease’s prognosis and having a negative impact on the patient’s quality of life.

It is essential to exclude other etiologies that lead to villous atrophy, namely Whipple’s disease, olmesartan enteropathy, eosinophilic gastroenteritis, enteropathy caused by human immunodeficiency virus, and giardia infection, among others [10]. In this case, the authors presented the systematic investigation developed to exclude all the possible differential diagnoses through an extensive and detailed approach, as described above.

The diagnosis of seronegative celiac disease is only definitively confirmed by clinical and histological improvement after twelve months on a gluten-free diet, although complete villi restitution can take up to three years [1].

Special attention should be paid to the development of possible complications during follow-up, such as hyposplenism, cancer (intestinal lymphoma, small bowel adenocarcinoma), and ulcerative jejunoileitis, which are more frequent in the elderly population [2].

## Conclusions

Celiac disease clinical manifestations can vary from light to severe, with marked symptoms and malabsorption that can lead to serious malnutrition and, ultimately, death. The absence of a positive result when searching for the antibodies can cause a delay in the diagnosis. The authors present this case due to its challenging management and to raise awareness for the clinical hypothesis of seronegative celiac disease in people presented with suggestive gastrointestinal or extraintestinal symptoms.
